# Graphene-Based Virus Enrichment Protocol Increases the Detection Sensitivity of Human Norovirus in Strawberry and Oyster Samples

**DOI:** 10.3390/foods13182967

**Published:** 2024-09-19

**Authors:** Shuqing Zhou, Min Jin, Jing Yin, Danyang Shi, Haibei Li, Zhixian Gao, Zhengshan Chen, Zhongwei Yang, Tianjiao Chen, Huaran Wang, Junwen Li, Dong Yang

**Affiliations:** Military Medical Sciences Academy, Academy of Military Sciences, Tianjin 300050, Chinajunwen9999@hotmail.com (J.L.)

**Keywords:** food contaminants, norovirus, rapid diagnosis, virus extraction

## Abstract

Human noroviruses (HuNoVs), the most prevalent viral contaminant in food, account for a substantial proportion of nonbacterial gastroenteritis cases. Extensive work has been focused on the diagnosis of HuNoVs in clinical samples, whereas the availability of sensitive detection methods for their detection in food is lacking. Here, we developed a virus enrichment approach utilizing graphene-based nanocomposites (CTAB-rGO-Fe_3_O_4_) that does not rely on large instruments and is suitable for on-site food pretreatment. The recovery efficiency of the developed virus enrichment procedure for serially diluted GII.4 norovirus ranged from 10.06 to 72.67% in strawberries and from 2.66 to 79.65% in oysters. Furthermore, we developed a real-time recombinase polymerase amplification (real-time RPA) assay, which can detect as low as 1.22 genome copies µL^−1^ of recombinant plasmid standard and has no cross-reactivity with genomes of astrovirus, rotavirus, adenovirus, and MS2 bacteriophage. Notably, the combined virus enrichment and real-time RPA detection assay enhanced the detection limits to 2.84 and 37.5 genome copies g^−1^ in strawberries and oysters, respectively, compared to those of qPCR. Our strategy, the graphene-based virus enrichment method combined with real-time RPA, presents a promising tool for sensitively detecting HuNoVs in food samples.

## 1. Introduction

Human noroviruses (HuNoVs) are the leading cause of acute gastroenteritis worldwide, accounting for approximately 699 million infections and over 200,000 estimated deaths per annum [1,2]. Among the various genotypes of HuNoVs, GII.4 is considered responsible for more than half of the outbreaks and sporadic infections in the past two decades [3,4]. In addition, a sudden increase and high prevalence of the GII.17 strain in Asian countries have occurred since 2014 [5,6]. Fecal–oral spread is generally the most important mode of transmission of HuNoVs [7]. The low infectious dose as well as the high stability outside a host allow HuNoVs to remain infectious in foods for a long period. Transmission through contaminated food might account for the rapid and extensive spread of disease outbreaks in closed settings. Therefore, a rapid and sensitive method for the detection of GII norovirus in food is vital for preventing and controlling viral outbreaks.

The general process for the detection of HuNoVs in food consists of virus extraction and molecular detection. During the virus extraction process, viral particles are separated from the food matrix and concentrated into a small volume, which is conducive to subsequent molecular detection. Frequently used methods for enriching HuNoVs include polyethylene glycol (PEG) precipitation, ultracentrifugation, ultrafiltration, immune concentration, and cationic separation [8,9,10,11]. The PEG precipitation method is inexpensive and easy to operate but requires incubation at 4 °C overnight to precipitate the virus, which can be a long time during acute outbreaks. Ultracentrifugation relies on expensive ultracentrifuges and requires trained professional operators. Ultrafiltration needs additional purification of the viral eluate to prevent the clogging of the filter. The immune-concentration method is based on specific antigen–antibody binding, which can specifically adsorb viruses. However, there are restrictions on how many samples can be processed, and the long-term storage of antibodies may reduce their activity. Cationic separation is based on electrostatic interactions between positively charged magnetic particles and negatively charged proteins on the surface of the viral capsid. When combined with a magnetic capture system, viral particles can be concentrated and purified from food matrices. Previously, a magnetic silica bead-based extraction protocol was reported to recover norovirus from spiked lettuce, with recovery yields ranging from 33 to 82% [10].

Previously, we synthesized a positively charged magnetic reduced graphene oxide composite (CTAB-rGO-Fe_3_O_4_) that can effectively enrich human enteric viruses [12]. The maximal loading of CTAB-rGO-Fe_3_O_4_ with GII.4 norovirus, human rotavirus (HRV), and human adenovirus (HAdV) reached 3.55 × 10^7^, 7.01 × 10^7^, and 2.21 × 10^7^ genome copies mg^−1^, respectively. Moreover, CTAB-rGO-Fe_3_O_4_ can be used to efficiently enrich these three viruses from complex aqueous samples such as seawater, surface water, and tap water. Therefore, the CTAB-rGO-Fe_3_O_4_-based cationic separation method offers great promise for the rapid and efficient enrichment of GII.4 norovirus from complex food matrices.

As for the molecular detection method, real-time reverse transcriptase polymerase chain reaction (real-time RT-qPCR) is the gold standard method for the detection of norovirus in food and clinical samples [13]. However, some limitations restrict its wide application at the point of care. For example, real-time RT-qPCR requires trained professionals in a specialized laboratory, and the amplification reaction requires precise instruments for large-scale fluorescence monitoring. In addition, the thermal cycling reaction process takes a long time, precluding rapid detection. Recombinase polymerase amplification (RPA) is a highly sensitive, specific, and fast isothermal nucleic acid amplification technology that has great application prospects in the field of on-site detection [14]. As a novel virus detection method, RPA has been applied to the rapid detection of dengue virus, Zika virus, duck hepatitis A virus, monkeypox virus, Crimean–Congo hemorrhagic fever virus, swine Japanese encephalitis virus, and mpox virus [15,16,17,18,19,20,21]. Accordingly, RPA technology can easily be adapted for rapid detection of norovirus in food.

In the present study, we describe a combined virus enrichment and detection method for the detection of GII.4 norovirus in strawberry and oyster samples. CTAB-rGO-Fe_3_O_4_ composites were first used as adsorbents to develop a GII.4 norovirus enrichment method for elution from strawberry and oyster samples. Meanwhile, a real-time RPA assay was developed for the detection of GII.4 norovirus. The combined virus extraction and real-time RPA detection assay were found to have satisfactory specificity, sensitivity, and capacity.

## 2. Materials and Methods

### 2.1. Viruses and Samples

The CTAB-rGO-Fe_3_O_4_ composite was synthesized and characterized according to our previous study [12]. Bacteriophage MS2, human rotavirus strain Wa, and human adenovirus type 41 were obtained from the American Type Culture Collection. Astrovirus (AstV) and norovirus genotypes (GII.3, GII.4, GII.6, and GII.17) were stored at −80 °C in our laboratory. Strawberry and oyster samples were purchased from Sanyizhuang Vegetable Market in Tianjin. Viral titers were expressed as genome copies (GC) per sample weight or volume.

### 2.2. Development of the Real-Time RPA Assay

#### 2.2.1. Primer and Probe Design 

The length of RPA primers is generally 30–36 bp, the GC content is between 20 and 70%, the melting temperature (T_m_) of the primer is between 50 and 100 °C, and the maximal allowable length of single mononucleotide repeat sequences is 5 bp. To ensure that the amplification reaction is completed in a short time, the optimum amplicon length is 100–200 bp. The conserved region in the ORF1-ORF2 junction gene of GII.4 norovirus was downloaded from the GenBank database (accession number: X86557.1) and used to construct the recombinant plasmid. The primers were designed using Primer-Blast “https://www.ncbi.nlm.nih.gov/tools/primer-blast/ (accessed on 18 August 2024)”. The probes were designed according to the primer screening results. The recombinant plasmid standard, primers, and probes were synthesized by Shanghai Sangon Biotech.

#### 2.2.2. Optimization of the Real-Time RPA Assay

The recombinant plasmid standard at a concentration of 1.22 × 10^4^ copies µL^−1^ was used as the template for the screening of primers and probes. The primer screening experiments were carried out using TwistAmp basic DNA amplification kit (TwistDx Inc., Cambridge, UK). A volume of 42.7 µL reaction solution was firstly prepared, containing 29.5 µL rehydration buffer, 11.2 µL deionized water, and 2 µL template. Then, 2.4 µL of 10 mM forward primer and 2.4 µL of 10 mM reverse primer were added to the reaction solution. Subsequently, the above mixture was mixed with lyophilized enzyme powder. After that, 2.5 µL magnesium acetate (280 mM) was added to the tops of reaction tube lids. The 50 µL reaction system was fully mixed and placed in a metal bath at 39 °C for 30 min. Finally, 5 µL of the reaction products were taken for agarose gel electrophoresis. Each reaction was conducted in triplicate, with ddH_2_O as a negative control.

The TwistAmpTM exo kit (TwistDx Inc., Cambridge, UK) was used to screen probes. Firstly, 46.9 µL reaction solution was prepared, containing 29.5 µL rehydration buffer, 2.1 µL of 10 mM forward and reverse primers, 11.2 µL deionized water, and 2 µL template. Then, 0.6 µL of 10 mM probe solution was added to the mixture. Subsequently, the above reaction solution was mixed with lyophilized enzyme powder. Finally, 2.5 µL magnesium acetate (280 mM) was added to the tops of reaction tube lids. The fluorescence intensity was recorded using a Lightcycler480 at 39 °C for 20 min. Each reaction was conducted in triplicate, with ddH_2_O as a negative control.

After the determination of the optimal group of primers and the probe, the temperature conditions for the real-time RPA reaction were optimized. Using the recombinant plasmid standard at a concentration of 1.22 × 10^4^ copies µL^−1^ as the template, the real-time RPA reactions were carried out at 37 °C, 39 °C, 40 °C, and 42 °C for 20 min at each temperature. Each reaction was conducted in triplicate, with ddH_2_O as a negative control.

#### 2.2.3. Specificity, Sensitivity, and Capability Analysis

The recombinant plasmid standard at a concentration of 1.22 × 10^4^ copies µL^−1^ was used as a positive control, and the nucleic acid samples extracted from different norovirus genotypes (GII.3, GII.6, and GII.17), AstV, MS2, HRV, and HAdV were used to determine the analytical specificity of the real-time RPA assay. Each reaction was conducted three times, with ddH_2_O as a negative control.

The recombinant plasmid standard was 10-fold serially diluted at concentrations of 1.22 × 10^0^–1.22 × 10^5^ copies µL^−1^. Then, 2 µL of each dilution was used as a template to perform the real-time RPA reaction at the optimal temperature for 20 min to determine the limit of detection (LOD). Each reaction was conducted three times with ddH_2_O as a negative control.

Thirty suspected norovirus samples (numbered 1–30) were used to test the practicability of the real-time RPA reaction system. The norovirus RNA was extracted using a QIAamp^®^ Viral RNA Mini Kit (QIAGEN, Hamburg, Germany) according to the manufacturer’s instructions. The extracted RNA was reverse-transcribed to cDNA and used as the template for real-time RPA and RT-PCR (see Supplementary Materials). Each reaction was conducted three times, with ddH_2_O as a negative control.

### 2.3. Detection of GII.4 Norovirus in Strawberry and Oyster Samples

#### 2.3.1. Preparation of Artificially Contaminated Strawberry and Oyster Samples

A GII.4 norovirus suspension was 10-fold serially diluted and stored at −80 °C for further use. Ten-fold serially diluted GII.4 norovirus was spiked in strawberry and oyster samples to determine the effect of inoculum level on recovery efficiency. To prepare the fruit sample, 25 g of strawberries was mixed with 100 µL of the serially diluted norovirus suspension and placed in a biosafety cabinet for 30 min. To prepare the seafood sample, 2.0 g of digestive glands of oysters was mixed with 100 µL of the norovirus suspension in a 50 mL centrifuge tube. The centrifuge tube was placed in a shaker and oscillated at 150 rpm and 37 °C for 30 min.

#### 2.3.2. Elution

Methods for the extraction of viruses from food depend on the food composition. Strawberries are a water-based food and are composed of carbohydrates. Oysters are considered shellfish foods, which accumulate and concentrate viral particles and other pathogens in the digestive system. The contaminated strawberries were directly washed with 25 mL of 0.7 M NaCl solution. For contaminated oysters, 2 mL of 10 mg mL^−1^ proteinase K solution was added to the samples and shaken at 150 rpm and 37 °C for 30 min. Then, 25 mL of 0.7 M NaCl was added, followed by shaking at 150 rpm and 37 °C for 15 min. After that, the mixture was centrifuged at 4000 rpm and 4 °C for 5 min.

#### 2.3.3. Enrichment of Norovirus

The obtained eluent was transferred to a 50 mL centrifuge tube with 1 mL of 3.2 mg mL^−1^ CTAB-rGO-Fe_3_O_4_ composite suspension. The centrifuge tube was placed horizontally in a constant temperature shaker and oscillated at 150 rpm and 37 °C for 60 min. Subsequently, an external magnet was applied for 3 min, after which the supernatant was discarded. The virus–composite conjugate was resuspended in 500 µL of sterile deionized water.

As a comparison, virus extraction was also performed according to the ISO/TS 15216-2:2019 standard method [22]. For strawberries, virus extraction was conducted by precipitation with PEG/NaCl. For oysters, viruses were extracted from the tissues of the digestive glands using proteinase K solution treatment. The resultant sample was subjected to virus RNA extraction followed by RT-PCR (see Supplementary Materials).

#### 2.3.4. Molecular Detection

The viral RNA was extracted using a QIAamp^®^ Viral RNA Mini Kit (QIAGEN, 52904) according to the manufacturer’s guidelines. The triol lysis method was used to extract norovirus RNA in the oyster sample. Then, 2 µL of the extracted RNA was reverse-transcribed to cDNA using the cDNA Synthesis Kit (TaKaRa, Dalian, China); the template (2 µL) was mixed with a random primer (1 µL), a dNTP mixture (1 µL), and RNase-free distilled water (6 µL), followed by incubation at 65 °C for 5 min. The mixture was cooled on ice for 5 min; then, 5 × PrimeScript II Buffer (4 µL), RNase inhibitor (0.5 µL, 40 U/µL), PrimeScript II RTase (1 µL, 200 U/µL), and RNase-free distilled water (4.5 µL) were added and treated at 30 °C for 10 min, 42 °C for 60 min, and 70 °C for 15 min.

The TaqMan qPCR assay was used to quantify the GC of HuNoV genotype GII.4. qPCR reaction mixtures contained 10 µL FastStart Universal Probe Master (Roche, Basel, Switzerland), 5 mM of each primer, 5 mM probe, 2 µL cDNA, and 6.5 µL RNase-free distilled water. Thermal cycling was performed at 50 °C (2 min) and 95 °C (10 min), and DNA was amplified with 40 cycles at 95 °C (15 s) and 60 °C (1 min). All qPCR reactions were performed in triplicate. RNase-free distilled water was used for negative controls.

Standard curves for HuNoV genotype GII.4 were set up by testing 10-fold serially diluted cDNA (Figure S1). qPCR was performed with a ViiA 7 Dx Real-Time PCR System (Applied Biosystems, Foster, CA, USA) using the primers and probes listed in Table S1. The qPCR assay was used to determine the recovery efficiency. Real-time RPA was used for qualitative detection of the virus in strawberry and oyster samples to evaluate the sensitivity of the assay. The fluorescence intensity was recorded using a T8-ISO (TwistDx Inc., Cambridge, UK) at 39 °C for 20 min.

### 2.4. Statistical Analysis

Data were analyzed using OriginPro 8.5. Each experiment was repeated three times. The recovery efficiency was calculated using Formula (1):

(1) $$Recovery\;efficiency\left(\%\right)=\frac{{GC}_{t}}{{GC}_{0}}\times 100$$

where GC_0_ is the initial virion concentration and GC_t_ is the amount of virions detected by real-time PCR.

## 3. Results and Discussion

### 3.1. Development of Real-Time RPA Detection Method

**Figure 1:**
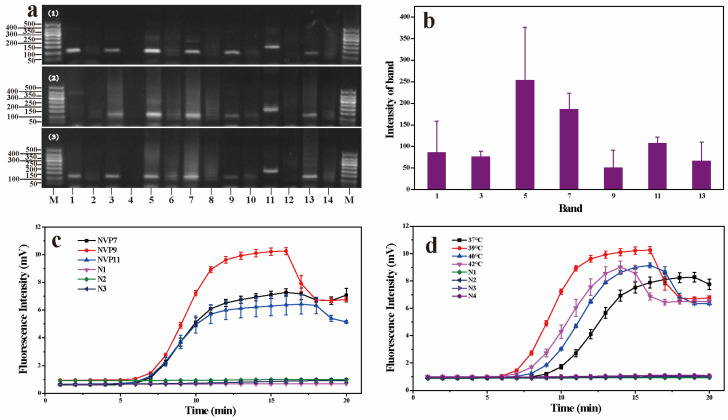
Agarose gel electrophoresis of real RPA products (**a**), intensity of the bands quantitated with ImageJ software (version 1.8.0) (**b**), probe screening (**c**), and reaction temperature optimization (**d**). (1), (2), and (3) are the results of three independent experiments, respectively. M: DNA molecular size standard DL50. Lanes 1, 3, 5, 7, 9, 11, and 13 are the positive reaction wells of the primer pairs NVF1/NVR1, NVF2/NVR2, NVF3/NVR3, NVF4/NVR4, NVF5/NVR5, NVF6/NVR6, and NVF7/NVR7, respectively. Lanes 2, 4, 6, 8, 10, 12, and 14 are the negative reaction wells of the primer pairs NVF1/NVR1, NVF2/NVR2, NVF3/NVR3, NVF4/NVR4, NVF5/NVR5, NVF6/NVR6, and NVF7/NVR7, respectively.

The primers and probes designed in this study are shown in Table S2. A total of seven primer pairs and three probes were designed. The results of primer screening are shown in Figure 1a,b. All seven primer pairs could effectively amplify the conserved specific fragment of norovirus. However, non-specific amplification was presented in lanes 3, 5, 7, and 13. The band intensity of lane 11 is higher than lane 1, and there was no primer dimer in the negative control reaction. Therefore, it was possible to design probes between the NVF6 and NVR6 primer binding sites.

The results of probe screening are shown in Figure 1c. All three primer–probe sets can produce exponential amplification curves, which are the positivity criteria of the RPA assay. When the NVP9 probe was used in the fluorescent RPA reaction system, the reaction initiation time was earlier than that of the probes NVP7 and NVP11, and the total fluorescence intensity was higher. There was no amplification reaction in the negative control. Therefore, the probe NVP9 showed better performance and was the most suitable for detecting the norovirus genome.

In order to determine the optimal reaction temperature of the real-time RPA reaction, the reaction was carried out at 37, 39, 40, and 42 °C for 20 min at each temperature. The experimental results are shown in Figure 1d. The results showed that when the reaction temperature was 39 °C, the reaction initiation time was earlier than at other temperatures, and the total fluorescence intensity was higher. There was no amplification reaction in the negative control, and the reaction could be completed at 16 min. Therefore, 39 °C was selected as the optimal reaction temperature in this study.

**Figure 2:**
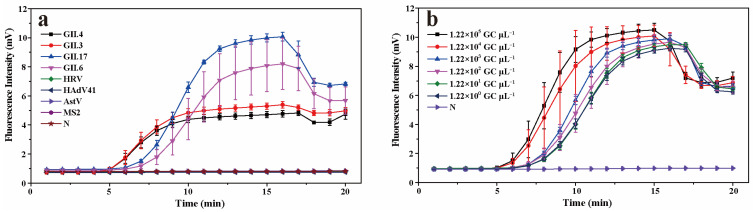
The specificity (**a**) and sensitivity (**b**) of the real-time RPA assay. N: negative control.

The specificity of the real-time RPA detection system is shown in Figure 2a. In order to determine the specificity of the real-time RPA reaction, the DNA or cDNA of norovirus genotypes (GII.3, GII.4, GII.6, and GII.17), AstV, MS2, HRV, and HAdV was used as templates for amplification under the optimal conditions. The results showed that norovirus genotypes (GII.3, GII.4, GII.6, and GII.17) had obvious amplification curves, while the fluorescence signals of AstV, MS2 phage, HRV, and HAdV did not change significantly with time. The results showed that the fluorescent RPA reaction established in this study could specifically amplify GII.3, GII.4, GII.6, and GII.17 norovirus, without amplifying AstV, MS2 phage, HRV, or HAdV.

In order to test the sensitivity of the system, the plasmid standard at concentrations of 1.22 × 10^5^, 1.22 × 10^4^, 1.22 × 10^3^, 1.22 × 10^2^, 1.22 × 10^1^, and 1.22 × 10^0^ GC µL^−1^ was used as a template for real-time RPA amplification. The results of real-time RPA sensitivity analysis are shown in Figure 2b, which indicated that the LOD of the real-time RPA method was 1.22 GC µL^−1^, and the time to reach the maximum fluorescence value was 17 min.

**Figure 3:**
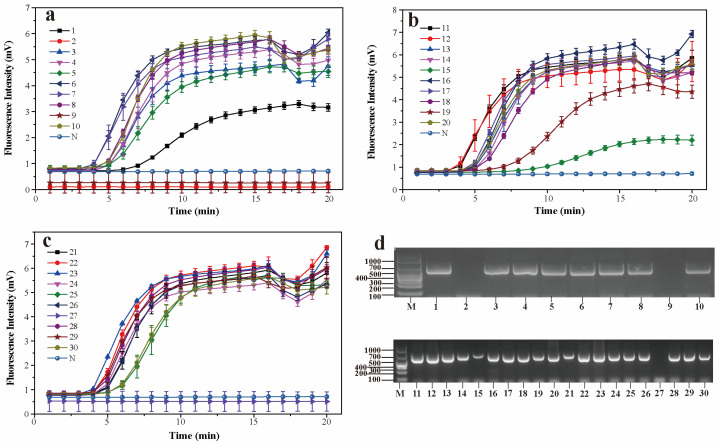
The performance of real-time RPA on 30 norovirus samples (**a**–**c**). RT-PCR results of 30 norovirus samples (**d**). M: DNA molecular weight standard DL2000; Lanes 1–30 are suspected norovirus samples. N: negative control.

The performance of real-time RPA on thirty suspected norovirus samples is shown in Figure 3 and Table S3. Among the thirty samples, twenty GII.3 norovirus samples and seven GII.4 norovirus samples were detected. Sample Nos. 2, 9, and 27 had no amplification signals, which was consistent with the results of RT-PCR (Figure 3d). The results showed that the real-time RPA assay could be used for the detection of GII.3 and GII.4 norovirus samples.

RPA is an isothermal nucleic acid amplification technique that can be carried out at room temperature with high sensitivity and specificity, and it was recently used to detect human norovirus. Moore and Jaykus [23] developed an RT-RPA assay targeting GII.4 New Orleans, with a sensitivity of 3.40 ± 0.20 log10 GC pre-reaction. Jia et al. [24] reported an RPA-based lateral flow strip assay for rapid detection of genogroup II noroviruses in the field. The assay can specifically detect pure GII noroviruses as well as RNA in boiled human feces samples with a sensitivity of 50 norovirus genome copies per reaction. Qian et al. [25] developed rapid, reliable, and portable detection systems for NOV genotype GII.4 by coupling reverse transcription recombinase polymerase amplification (RT-RPA) with CRISPR-Cas12a (RT-RPA-Cas12a). These methods focused particularly on norovirus detection in fecal samples, while not verifying the applicability in food samples, which are the main transmission vector of human norovirus. Han et al. [26] established a rapid and reliable reverse transcription recombinase polymerase amplification (RT-RPA) method for the detection of NoV GII, with a detection limit as low as 1.66 × 10^2^ GC µL^−1^. The assay was tested using real samples, including food, water, and feces. However, only one kind of food (digestive glands in the shellfish) was tested.

### 3.2. Recovery Efficiency of Graphene-Based Norovirus Enrichment Assay 

To simulate contaminated produce, seven serially diluted GII.4 norovirus suspensions (10^6^ to 10^0^ GC) were used to contaminate strawberries, as shown in Table 1. When the virus amount was decreased from 10^6^ to 10^2^ GC, the virus recovery efficiency increased, reaching 10.06%, 11.48%, 21.25%, 55.63%, and 72.67%, respectively. However, the genome could not be detected at the lowest concentrations of 10^1^ and 10^0^ GC using qPCR.

To simulate contaminated seafood, oysters were mixed with five serially diluted virus suspensions (10^4^ to 10^0^ GC). As shown in Table 1, when the virus amount was decreased from 10^4^ to 10^1^ GC, the virus recovery efficiency increased, reaching 2.66%, 11.32%, 24.97%, and 79.65%, respectively. However, the virus could not be detected at the concentration 10^0^ using qPCR.

By using PEG, Stals obtained 20.7–47.7% recoveries when extracting 104 and 106 GII norovirus genomic copies from 10 g of strawberries [27]. Benefiting from the adsorption ability of graphene and the attractive electrostatic interactions, the negatively charged viral particles can be efficiently absorbed on the positively charged CTAB-rGO-Fe_3_O_4_ composites. From the data in Table 1, it is apparent that norovirus recovery efficiency in strawberries and oysters was increased with the decrease in the level of the virus inoculum. This result is consistent with the finding of a study by Fumian et al. [28], who reported that the recovery increased from 5.2 to 72.3% and 6 to 56.3% for lettuce and cheese samples, respectively, with a decrease in the level of the virus inoculum. Moreover, the nanocomposite-based virus extraction protocol significantly improves the detection limit of qPCR. This apparent improvement in detection limits can be attributed to two aspects. On the one hand, during the virus extraction process, viral particles are separated from the food matrix and concentrated to a small volume, and qPCR inhibitory compounds are removed. On the other hand, the virus elution buffer used in this study was 0.7 M NaCl solution, which helps the CTAB-rGO-Fe_3_O_4_ composite to absorb the virus from the food elution buffer. The result is consistent with our previous study.

### 3.3. Performance of Graphene-Based Virus Extraction Protocol Combined with Real-Time RPA

**Figure 4:**
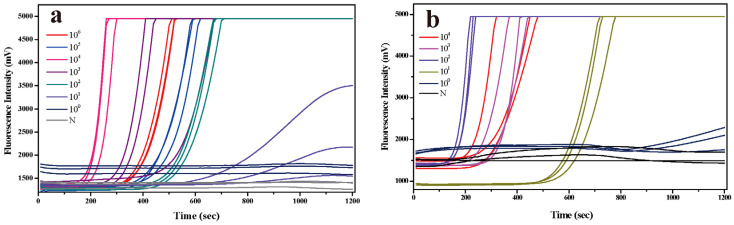
Performance of real-time RPA on artificially contaminated strawberry (**a**) and oyster (**b**) samples. N: negative control.

For artificially contaminated strawberries, real-time RPA was used to detect nucleic acids obtained from seven dilution gradients. The results are shown in Figure 4a. The results showed that there were fluorescence amplification signals in six gradients with a virus amount of 10^6^–10^1^ GC, indicating that the real-time RPA assay combined with the CTAB-rGO-Fe_3_O_4_ composite-based virus extraction protocol could detect norovirus concentrations as low as 2.84 GC g^−1^ in strawberries. Real-time RPA is more sensitive than quantitative PCR, easier to operate, and less time-consuming, which is conducive to fast and accurate detection.

For artificially contaminated oysters, the nucleic acid obtained from 10^4^–10^0^ GC dilutions was detected by real-time RPA, as shown in Figure 4b. Four gradient dilutions with a virus amount of 10^4^–10^1^ GC resulted in fluorescent amplification signals, which proved that the real-time RPA combined with the CTAB-rGO-Fe_3_O_4_ composite could detect norovirus at concentrations as low as 37.5 GC g^−1^ in oysters, which was consistent with the quantitative PCR method.

A comparison of detection limits between the ISO/TS 15216-2:2019 standard method and this study is shown in Table 2. For the ISO/TS 15216-2:2019 standard method, 25 g of strawberries was spiked with 71 GC of GII.4 norovirus and precipitated with PEG/NaCl, and 2 g of oyster glands was spiked with 75 GC of GII.4 norovirus and digested using proteinase K solution. The results show that no virus was detected using the ISO/TS 15216-2:2019 standard method. Compared with the ISO/TS 15216-2:2019 standard method, the CTAB-rGO-Fe_3_O_4_ composite-based virus extraction protocol apparently results in a lower detection limit.

The existing food pretreatment procedures for virus extraction and purification are quite tedious, which hinders the rapid and sensitive diagnosis of norovirus in food. The combined virus extraction and detection assay established in this study has good specificity, high sensitivity, and good reproducibility. It can rapidly and accurately detect GII.4 norovirus and can be widely used in food monitoring departments. It is promising for the rapid and sensitive detection of norovirus in food samples.

## 4. Conclusions

In the present study, we developed a combined graphene-based virus extraction protocol and real-time RPA detection method for GII norovirus in strawberry and oyster samples. The real-time RPA detection method has good specificity for norovirus, high sensitivity, and good reproducibility. It can be used to detect GII.3, GII.4, GII.6, and GII.17 norovirus with a detection limit as low as 1.22 GC µL^−1^. The combined detection method was able to detect GII.4 norovirus at concentrations as low as 2.84 and 37.5 GC g^−1^ in strawberries and oysters, respectively. This method can be used to analyze food samples suspected of being infected or contaminated with norovirus, providing great help for implementing appropriate control measures to reduce the spread of the virus and the magnitude of outbreaks.

## Figures and Tables

**Table 1:** Recovery rates of GII.4 norovirus from artificially contaminated strawberry and oyster samples.

Food Samples	Inoculation Level of Inoculum (×10^2^ Genome Copies)	Detection by qPCR (mean ± SD) (×10^2^ Genome Copies)	Recovery Efficiency (%)
Strawberry (25 g)	11,614.23	1168.96 ± 167.70	10.06
	1147.00	131.62 ± 44.03	11.48
	296.81	63.07 ± 5.99	21.25
	45.47	25.29 ± 1.94	55.63
	8.99	6.54 ± 0.65	72.67
	0.71	nd ^a^	nd
	0.05	nd	nd
Oyster (2.0 g)	140.82	3.75 ± 0.44	2.66
	37.12	4.20 ± 0.72	11.32
	9.82	2.45 ± 0.50	24.97
	0.75	0.60 ± 0.08	79.65
	0.08	nd	nd

^a^ nd = not determined.

**Table 2:** Comparison of detection limits of GII.4 norovirus extraction methods for strawberry and oyster.

Protocol	Food Matrix	Detection Limit	Reference
Graphene-based virus enrichment protocol	Strawberry (25 g)	2.84 GC g^−1^	This study
	Oyster (2 g)	37.5 GC g^−1^	This study
ISO/TS 15216-2	Strawberry (25 g)	nd ^a^	This study
	Oyster (2 g)	nd	This study

^a^ nd = not determined.

## Data Availability

The data presented in this study are available on request from the corresponding author. The data are not publicly available due to privacy.

## Supplementary Materials

The following supporting information can be downloaded at https://www.mdpi.com/article/10.3390/foods13182967/s1: Figure S1: Standard curve of qPCR for cDNA of HuNoV genotype GII.4; Table S1: Primers and probes for detection of HuNoV; Table S2: Primers and probes for real-time RPA; Table S3: The results of real-time RPA and RT-PCR on thirty suspected norovirus samples. Refs. [29,30,31,32] are cited in Supplementary Materials.
