# Supplementary material for: Graphene-Based Virus Enrichment Protocol Increases the Detection Sensitivity of Human Norovirus in Strawberry and Oyster Samples
Source: Foods. 2024 Sep 19;13(18):2967. doi: 10.3390/foods13182967 (PMC11431745; doi:10.3390/foods13182967)
Supplement: Supplementary file 1 [file foods-13-02967-s001.zip › foods-3170043-supplementary.pdf]

# Graphene-Based Virus Enrichment Protocol Increases the Detection Sensitivity of Human Norovirus in Strawberry and Oyster Samples

**Shuqing Zhou †, Min Jin †, Jing Yin †, Danyang Shi, Haibei Li, Zhixian Gao, Zhengshan Chen, Zhongwei Yang, Tianjiao Chen, Huaran Wang, Junwen Li and Dong Yang ***

Military Medical Sciences Academy, Academy of Military Sciences, Tianjin 300050, China

* Correspondence: yangd8611@163.com

† These authors contributed equally to this work.

## Supplementary material

### 1. Detection of fecal samples

The fecal specimens were resuspended with PBS and gently oscillated on a vortex oscillator until the feces were dispersed. The feces were centrifuged at 12, 500 rpm for 2 min at 4°C, and the supernatant was filtered with a 0.22 μm filter to remove bacteria and transferred to labeled EP tubes.

RT-PCR was performed to determine whether the fecal samples were clinically infected with norovirus. RT-PCR reaction mixtures contained 15 μL Premix Taq (TaKaRa Taq™ Version 2.0 plus dye), 15 mM of each primer, 3 μL cDNA, and 9 μL RNase-free distilled water. Thermal cycling was performed at 95°C (15 min), and DNA was amplified with 40 cycles at 95°C (1 min), 50°C (1 min) and 72°C (1 min), followed by incubation at 72°C (10 min) and hold at 4°C. All PCR reactions were performed in triplicate. RT-PCR was performed with a thermal cycler (Applied Biosystems, USA) using the primer set (MON431/G2SKR) listed in Table S1.

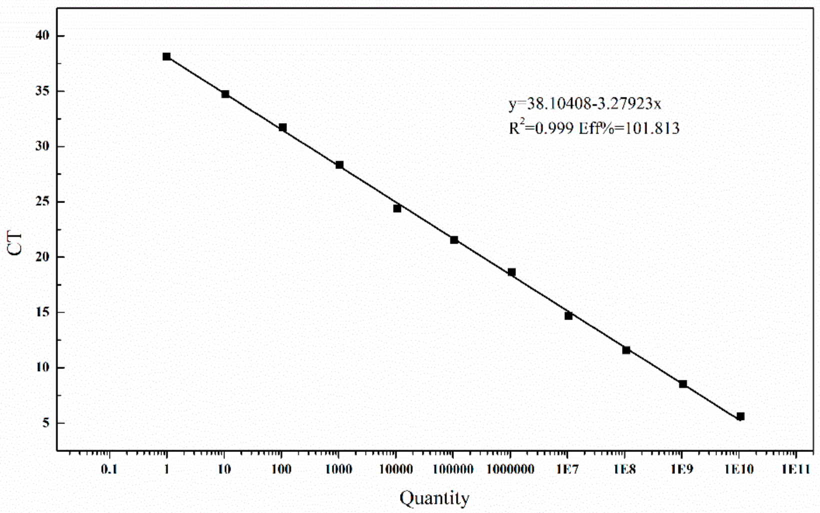


Figure S1. Standard curve of qPCR for cDNA of HuNoV genotype GII.4.

Table S1. Primers and probes for detection of HuNoV.

| Primers or probe | Sequence (5'-3') | Product length (bp) | References |
|---|---|---|---|
| QNIF2d | ATGTTCAGRTGGATGAGRTTCTCWGA | 89 | (Stals et al., 2012) |
| COG2R | TCGACGCCATCTTCATTCACA | | |
| QNIFS | FAM-AGCACGTGGGAGGGCGATCG-TAMRA | | |
| MON431 | TGGACIAGRGGICCYAAYCA | 557 | (Anderson et al., 2001; Kojima et al., 2002; Shinohara and Kageyama, 2002) |
| G2SKR | CARGARBCNATGTTYAGRTGGATGAG | | |

Table S2. Primers and probes for real-time RPA.

| Primers /Probes | sequence（5'-3'） | Product length（bp） |
|---|---|---|
| NVF1 | CRGCATTCTAYAGCAAAATYAGYAAGYTRGTCA | 107 |
| NVR1 | GAATCTCATCCATCTGAACATTGGYTCTTGTCT | |
| NVF2 | CATTCTAYAGCAAAATYAGYAAGYTRGTCA | 106 |
| NVR2 | GAGAATCTCATCCATCTGAACATTGGYTCTT | |
| NVF3 | AGCATTCTAYAGCAAAATYAGYAAGYTRGTCA | 107 |
| NVR3 | AGAATCTCATCCATCTGAACATTGGYTCTTGTC | |
| NVF4 | GCATTCTAYAGCAAAATYAGYAAGYTRGTCA | 107 |
| NVR4 | GAGAATCTCATCCATCTGAACATTGGYTCTTGT | |
| NVF5 | CCRGCATTCTAYAGCAAAATYAGYAAGYTRGTCA | 107 |
| NVR5 | AATCTCATCCATCTGAACATTGGYTCTTGTCTK | |
| NVF6 | YAGCAAAATYAGYAAGYTRGTCATTGCAGARY | 170 |
| NVR6 | GTCAYTCGACGCCATCTTCATTCACAAARC | |
| NVF7 | GGYCCRGCATTCTAYAGCAAAATYAGYAAG | 111 |
| NVR7 | GAATCTCATCCATCTGAACATTGGYTCTTGTC | |
| NVP7 | GGATTTTTACGTGCCCAGACAAGAGCCAA/i6FAMdT//idSp/T/iBHQ1dT/CAGATGGATGAGA/iSpC3/ | - |
| NVP9 | CGTGCCCAGACAAGAGCCAATGTTCAGA/i6FAMdT/G/idSp/A/iBHQ1dT/GAGATTCTCAGAT/iSpC3/ | - |
| NVP11 | GACAAGAGCCAATGTTCAGATGGATGAGA/i6FAMdT//idSp/C/iBHQ1dT/CAGATCTGAGCAC/iSpC3/ | - |

Table S3. The results of real-time RPA and RT-PCR on thirty suspected norovirus samples.

| Samples | Viral agent | Results | |
|---|---|---|---|
| | | RT-PCR | Real-time RPA |
| 1 | GII.3 | P[a] | P |
| 2 | nd[b] | N[c] | N |
| 3 | GII.4 | P | P |
| 4 | GII.3 | P | P |
| 5 | GII.3 | P | P |
| 6 | GII.3 | P | P |
| 7 | GII.3 | P | P |
| 8 | GII.3 | P | P |
| 9 | nd | N | N |
| 10 | GII.4 | P | P |
| 11 | GII.3 | P | P |
| 12 | GII.3 | P | P |
| 13 | GII.3 | P | P |
| 14 | GII.4 | P | P |
| 15 | GII.3 | P | P |
| 16 | GII.4 | P | P |
| 17 | GII.4 | P | P |
| 18 | GII.3 | P | P |
| 19 | GII.3 | P | P |
| 20 | GII.3 | P | P |
| 21 | GII.3 | P | P |
| 22 | GII.3 | P | P |
| 23 | GII.3 | P | P |
| 24 | GII.4 | P | P |
| 25 | GII.3 | P | P |
| 26 | GII.4 | P | P |
| 27 | nd | N | N |
| 28 | GII.3 | P | P |
| 29 | GII.3 | P | P |
| 30 | GII.3 | P | P |

[a] P=positive, [b] nd =not determined, [c] N=negative.

## References


1. Anderson, A.D., Garrett, V.D., Sobel, J., Monroe, S.S., Fankhauser, R.L., Schwab, K.J., Bresee, J.S., Mead, P.S., Higgins, C., Campana, J., Glass, R.I. (2001). Multistate outbreak of Norwalk-like virus gastroenteritis associated with a common caterer. *Am J Epidemiol, 154,* 1013-1019. https://doi.org/10.1093/aje/154.11.1013.
2. Kojima, S., Kageyama, T., Fukushi, S., Hoshino, F.B., Shinohara, M., Uchida, K., Natori, K., Takeda, N., Katayama, K. (2002). Genogroup-specific PCR primers for detection of Norwalk-like viruses. *J Virol Methods, 100,* 107-114. https://doi.org/10.1016/s0166-0934(01)00404-9.
3. Shinohara, M., Kageyama, T. (2002). Rapid and efficient detection method of Norwalk virus. *Nihon Rinsho, 60,* 1181-1187.
4. Stals, A., Mathijs, E., Baert, L., Botteldoorn, N., Denayer, S., Mauroy, A., Scipioni, A., Daube, G., Dierick, K., Herman, L., Van Coillie, E., Thiry, E., Uyttendaele, M. (2012). Molecular detection and genotyping of noroviruses. *Food and environmental virology, 4,* 153-167. https://doi.org/10.1007/s12560-012-9092-y.
